# CuReSim-LoRM: A Tool to Simulate Metabarcoding Long Reads

**DOI:** 10.3390/ijms241814005

**Published:** 2023-09-12

**Authors:** Yasmina Mesloub, Delphine Beury, Félix Vandermeeren, Ségolène Caboche

**Affiliations:** Univ. Lille, CNRS, Inserm, CHU Lille, Institut Pasteur de Lille, US 41-UAR 2014-PLBS, F-59000 Lille, France

**Keywords:** read simulation, metabarcoding, long reads, benchmark

## Abstract

Metabarcoding DNA sequencing has revolutionized the study of microbial communities. Third-generation sequencing producing long reads had opened up new perspectives. Obtaining the full-length ribosomal RNA gene would permit one to reach a better taxonomic resolution at the species or the strain level. However, Oxford Nanopore Technologies (ONT) sequencing produces reads with high error rates, which introduces biases in analysis. Understanding the biases introduced during the analysis allows one to better interpret the biological results and take care of conclusions drawn from metabarcoding experiments. To benchmark an analysis process, the ground truth, i.e., the real composition of the microbial community, has to be known. In addition to artificial mock communities, simulated data are often used to evaluate the biases and performances of the bioinformatics analysis step. Currently, no specific tool has been developed to simulate metabarcoding long reads, mimic the error rate and the length distribution, and allow one to benchmark the analysis process. Here, we introduce CuReSim-LoRM, for the customized read simulator to generate long reads for metabarcoding. We showed that CuReSim-LoRM is able to produce reads with varying error rates and length distributions by mimicking the real data very well.

## 1. Introduction

Metabarcoding provides a rapid means of biodiversity assessment by identifying multiple taxa simultaneously in a sample using DNA sequencing. Molecular markers conserved and shared across various taxonomic groups are used to assess the microbiota composition. For bacterial species, the 16S rRNA gene is widely used as it contains conserved regions in which the PCR can be initiated and nine hyper-variable regions that allow for the discrimination of taxa [1]. Metabarcoding has become a routine application with the development of high-throughput short-read sequencing [2]. Indeed, several hundreds of samples can be sequenced with a single run of benchtop sequencers, allowing one to obtain the microbial composition and the taxonomic abundance. With short reads, a maximum of 500 bp-regions can be sequenced, representing two or three hyper-variable regions, allowing one to reach, at best, the genus level and, for some bacterial taxa, not allowing one to discriminate between them [3]. The arrival of third-generation sequencing producing (ultra)-long reads has opened up new perspectives in the sequencing field. For example, the Oxford Nanopore Technologies (ONT) company offers portable to ultra-high throughput scalability with real-time data delivery and the ability to produce short to ultra-long fragments of native DNA or RNA. The production of long reads entirely redesigns the metabarcoding application by allowing one to sequence the complete 16S rRNA gene and even the complete ribosomal operon. Obtaining the full-length 16S rRNA gene would permit one to reach a better taxonomic resolution at the species or the strain level. However, the ONT third-generation sequencing produces reads with high error rate compared to short-read sequencing. Indeed, the error rate of ONT sequencing is between 1% and 15%, depending on the flowcell version, the basecaller, and the kit chemistry. In metabarcoding experiments, the observed values are between 8% and 15% [4,5]; these high error rates will introduce biases in analysis.

Understanding the biases introduced during the analysis allows one to better interpret the biological results and take care of conclusions drawn from metabarcoding experiments. To benchmark an analysis process, the ground truth, i.e., the real composition of the microbial community, needs to be known. To achieve this goal, a mock community can be used. Indeed, using an artificial community in which the taxa and their abundance are known allows one to compute metrics such as true positives (reads assigned to a correct taxon) and false positives (reads assigned to an incorrect taxon). However, this kind of biological community does not permit one to discriminate between true negatives (i.e., reads that have to be unassigned) and false negatives (i.e., reads not assigned but that should have been assigned). To fill this gap, simulated data can be used. Read simulators are widely used in bioinformatics to benchmark biases in analysis processes, to evaluate new tools, or to optimize parameters. In metabarcoding studies, biases can be introduced during the sequencing library preparation, for example, during the amplification step, but also during the bioinformatics analysis, for example, biases due to the databases and/or the algorithmic methods. In addition, the biases in taxonomic abundances are also well known in metabarcoding studies and can be evaluated with simulated data. Lots of read simulators have been developed since they are always dedicated to a given sequencing technology and a set of applications [6].

Several publications were focused on the analysis of 16S ONT metabarcoding long reads. In Urban et al. [7], the authors provided optimized experimental and bioinformatics guidelines, including a benchmark with twelve taxonomic classification tools for ONT metabarcoding data, using a mock community to benchmark the analysis step. Emu is an approach that uses an expectation-maximization algorithm to generate taxonomic abundance profiles from full-length 16S rRNA reads [8]. The authors used simulated data obtained with DeepSimulator [9] and mock communities to show that Emu is capable of accurate microbial community profiling. In Manske et al. [10], the authors introduced a tool called MetaG with an intuitive web interface that makes the software accessible to a vast range of users. Evaluation of MetaG’s performances was performed on real and simulated data produced with NanoSim [11]. DeepSimulator and NanoSim, two ONT read simulators, were used in a 16S metabarcoding context but were not developed and were not validated for this task. They are not able to directly generate reads from several genomes with abundance profiles. In addition, the error model and the read length distribution were not compared to real 16S full-length data.

Here, we introduced CuReSim-LoRM, for the customized read simulator to generate Long Reads for Metabarcoding. CuReSim-LoRM is based on CuReSim, a short-read simulator that we have previously developed [12]. Our new simulator is able to produce metabarcoding reads with ONT error and read-length profiles. We first compared simulated data produced with CuReSim-LoRM and other simulators to real data. We then tested CuReSim-LoRM on several datasets with varying error rates and length distributions. All the results showed that CuReSim-LoRM is able (i) to produce simulated reads showing an error profile very close to the real data, (ii) to produce a read length distribution mimicking the real one, and (iii) to produce a wide range of data with varying error rates and length distributions. To our knowledge, CuReSim-LoRM is the first tool able to simulate ONT metabarcoding reads.

## 2. Results

### 2.1. Comparison of NanoSim-H, DeepSimulator, and CuReSim-LoRM Simulated Data with Real Data

Simulated reads were obtained for the newLot dataset with NanoSim-H, DeepSimulator, and CuReSim-LoRM (see Section 4 for more details). Three datasets were simulated with NanoSim-H: dataset1 corresponds to the use of −circular option as in [10], dataset2 was obtained without the −circular option, and dataset3 was generated by training the error model with the newLot data and the option −circular. DeepSimulator was used with default parameters as done in [8]. The error model used is the default context independent pore model as authors do not recommend users to train customized pore models.

**Figure 1:**
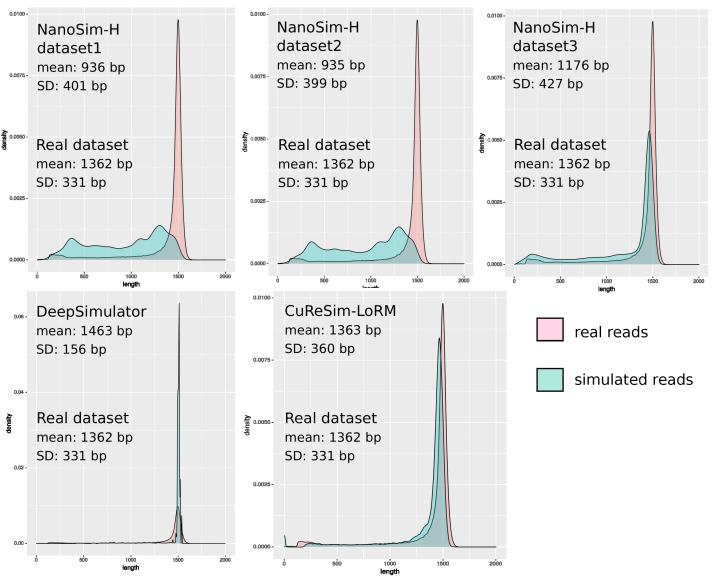
Read length distribution of real and simulated data for the newLot dataset. The distribution of the simulated read lengths is shown in blue and in light red for the real reads. For each simulation, the mean read length and the standard deviation (SD) are given.

Figure 1 shows the read length distribution of simulated reads compared to real reads. The mean length for the real dataset was 1362 bp, with a standard deviation (SD) of 331 bp. For NanoSim-H, only dataset3 simulated with the error model trained with the real newLot dataset drew up the real length distribution, with a mean length of 1176 bp and an SD of 427 bp. DeepSimulator did not manage to simulate read length close to the real data. Finally, CuReSim-LoRM was the closest to the real length distribution, with a mean length of 1363 bp and a SD of 360 bp.

Table 1 shows the metrics obtained with simulated data compared to the real newLot data (see Section 4 for more details about the metric computation). The real data showed an error rate of 14.45%. CuReSim-LoRM was the closest, with an error rate of 14.50%. For NanoSim-H, the error rates varied from 12.94 to 18.77% depending on the dataset. DeepSimulator showed an error rate of only 10.84%. Mapping reads with minimap2 against the ZymoBIOMICS reference sequences led to 3.4% of unmapped reads in the real dataset. Once more, CuReSim-LoRM showed the closest value with 5.5% of unmapped reads and DeepSimulator showed the furthest value with only 0.01% of unmapped reads. The percentage of identity and the standard deviation obtained with bbmap showed that the furthest results were obtained with DeepSimulator and the closest with CuReSim-LoRM. For NanoSim-H, dataset3 showed the lowest identity percentage, which was in agreement with the highest error rate observed before. The precision and recall values obtained from the taxonomy assignment against the ZymoBIOMICS reference sequences with minimap2 were then computed. The precision values were equal to 1 for all simulation datasets, except for the NanoSim-H dataset1, for which it was equal to 0.99. The recall value was of 0.96 with the real dataset and was between 0.9 for the NanoSim-H dataset3 and 1 for DeepSimulator, meaning that DeepSimulator did not manage to simulate reads that were not assigned or miss-assigned such as in the real dataset. A taxonomy assignment against the rrnDB database returned a precision of 0.94 and a recall of 0.91 for the real data. Similar values were obtained with the CuReSim-LoRM reads, with a value of 0.91 and 0.88, respectively. The precision was lower for other simulators, between 0.84 and 0.87, explained by an increase in false positives. Using the silva database, the precision and recall values were the lowest for the real dataset, with 0.74 and 0.72, respectively, probably because the silva database contains many more sequences than other databases and the probability to assign a wrong species increases. The three NanoSim-H datasets showed the lowest values: 0.62, 0.64, and 0.67 for precision and 0.55, 0.59, and 0.6 for recall (resp. dataset1, dataset2, and dataset3), whereas DeepSimulator showed a value of 0.73 for both precision and recall, explained by a very low number of unmapped reads with this simulator. The precision and recall obtained with CuReSim-LoRM were close to the real values, with a precision of 0.76 and a recall of 0.72.

All of these results have shown that CuReSim-LoRM is able to better simulate metabarcoding long reads than other simulators, with the newLot dataset representing a standard ONT run and showing read features close to the real ones. CuReSim-LoRM was then evaluated with several datasets showing a wide range of error rates and read length distributions.

### 2.2. Evaluation of CuReSim-LoRM with Challenging Datasets

In this part, CuReSim-LoRM was evaluated with five more challenging datasets. Indeed, these datasets showed a wide range of error rates and read length distributions (see Section 4 for the description of the datasets).

**Figure 2:**
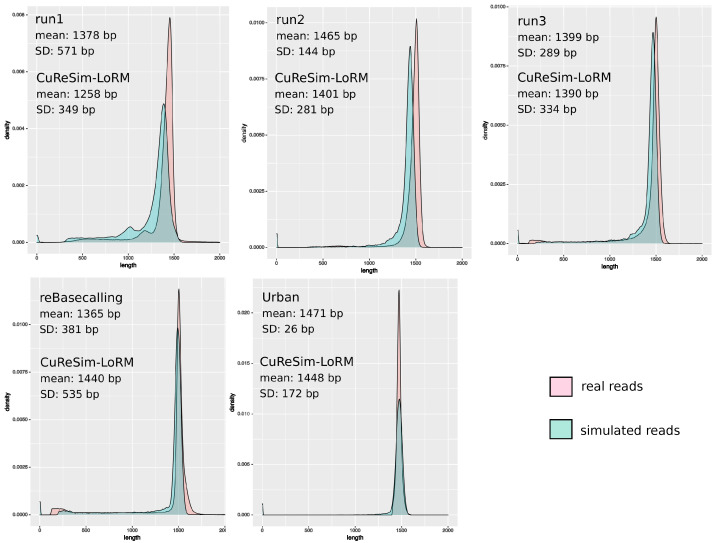
Read length distribution comparing simulated and real datasets. The distribution of the simulated read lengths is shown in blue and in light red for the real reads. For each simulation, the mean read length and the standard deviation (SD) are given for real and CuReSim-LoRM data.

Figure 2 shows the read length distribution of real and simulated reads. The run1 was the first run produced in our laboratory in 2017, with an older version of kit chemistry. The profile of this run was very different from the others, with the smallest mean length equal to 1258 bp and the largest standard deviation of 571 bp. A small peak at 1200 bp, in addition to the main peak at 1450 bp, appeared in the read length distribution. CuReSim-LoRM was able to fit the original length distribution with a mean length of 1258 bp and a SD of 349 bp, but the distribution was slightly shifted to the left. The run1 dataset, produced with an older chemistry and basecaller, might contain more noisy reads than other runs. Noisy reads, showing random sizes, are more difficult to simulate, which could explain the observed slight difference between real and simulated reads. Run2 and run3 showed similar profiles, with the mean length of run2 greater than run3 and a smaller SD. The simulated reads showed very close profiles and values to the real data for run2 and run3. The CuReSim-LoRM reads simulated from the reBasecalling data showed a similar length distribution, but the SD was higher in simulated data with 535 bp, whereas the SD was equal to 381 bp in the real data. Finally, the Urban run is particular because the authors kept only reads with lengths between 1.4 kb and 1.6 kb. This filter implies a high mean length of 1471 bp and a very low SD of 26 bp. By manually tuning the CuReSim-LoRM parameters, a similar distribution of the read length was obtained with a mean length of 1448 bp, but the SD of simulated data was higher (172 bp) than the value observed in real data.

Table 2 shows the metrics obtained for simulated data generated with CuReSim-LoRM compared to the real datasets. The error rates obtained with CuReSim-LoRM were very close to the real values. Our simulator was able to generate reads with error rates varying from 10.6% to 17.4%. The percentages of unmapped reads followed the real percentages, except for run2, where only 0.7% of reads were unmapped in real data, but a percentage of 4.9% was observed with simulated reads. The percentages of identity and the standard deviations computed with bbmap were very close to the values obtained with real data. The precision and recall values were very similar to the values observed with the real data, whatever the database used. The furthest values were obtained with the Urban dataset, especially against the silva database. Indeed, the precision and recall values for this dataset were very low (0.69 for both precision and recall with real data), but simulated data showed a precision of 0.75 and a recall of 0.74, with more false positives in the real data.

In this part, we showed that CuReSim-LoRM is able to simulate metabarcoding data with varying error rates and length distributions by mimicking the real data.

## 3. Discussion

In this paper, we introduced CuReSim-LoRM, a new tool to simulate long reads for metabarcoding. Our new simulator was compared to two other long read simulators and was evaluated on six datasets. To evaluate how well the simulated data mimics the real data properties, we used several metrics. First, the read length distribution of simulated and real data were plotted to see how well the distribution of simulated data fits the real one. Then, we computed the error rate, the percentage of unmapped reads, and the precision and recall values from the assignment of reads against three databases for real and simulated datasets. We hypothesized that if these values were similar between real and simulated data, then we can consider that simulated data mimics the real data well. In this evalution protocol, neither empirical values nor statistical tests were available to decide if the simulated data are really close to the real data. However, we first compared the results of CuReSim-LoRM to two other simulators that were already used in metabarcoding evaluation studies and showed that the metrics obtained with our simulator were the closest to the real data. We then evaluated CuReSim-LoRM on five additional datasets, showing a wide range of error rates, ranging from 10.5% to 17.4%, and heterogeneous read length distribution. The difference between the error rates from CuReSim-LoRM and real data was between 0.01% and 0.44%. The differences between the recall and precision values ranged from 0 to 0.06. These values can be considered as low, meaning that CuReSim-LoRM is able to simulate similar read behavior better than the real one. Concerning the study of the read length, the results showed that CuReSim-LoRM produced reads with length distribution well fitted to the distribution of the real data, except for the run1 dataset. This sequencing run was produced with an older chemistry and basecaller and might contain more noisy reads than other runs. Noisy reads, showing random sizes, are more difficult to simulate. However, the results presented here have shown that CuReSim-LoRM is able to train and simulate a very heterogeneous error model, being the only tool able to simulate reads mimicking real ONT metabarcoding data. The ability to train new error models allows CuReSim-LoRM to follow future technical updates.

Working with simulated data allows one to formally evaluate the bioinformatics analysis step, new tools, and databases but also the taxonomic abundance bias, which can be introduced during the analysis. In this article, we worked with the 16S rRNA gene, the specific marker of bacteria. However, our tool is able to produce simulated reads from any amplicon sequencing data such as 18S rRNA, the taxonomic marker of eukaryotes, or as ITS for fungi. The introduction of CuReSim-LoRM will help scientists to better understand biases in bioinformatics analysis and databases and will further the improvement of support of metabarcoding long reads.

## 4. Materials and Methods

### 4.1. Datasets

The ZymoBIOMICS Microbial Community Standard (Zymo Research, D6305) contains eight bacteria (3 Gram-negative and 5 Gram-positive) and two yeasts. This mock community was used in several studies to benchmark ONT sequencing technology and tools for 16S metabarcoding application [7,13]. We also used this mock community in our experiments, with two different lots: lot V1 (ZRC187324) and lot V2 (ZRC190812). Between the two lot versions, five strains were replaced in the community with similar strains. Table 3 shows the datasets used in this study.

We produced 3 sequencing runs with Lot V1 named run1, run2, and run3 and one run with lot V2 named newLot. The basecalling step was performed with Albacore for run1 and run2, and guppy was performed for the other runs. The newLot data was rebasecalled using the high accuracy model (HAC) of guppy, named the reBasecalling dataset in this study. We added an external dataset, available on SRA (ERR3806867), consisting of the sequencing of the same ZymoBIOMICS community with ONT technology and named Urban in Table 3. Note that this dataset does not contain the raw data: all reads shorter than 1.4 kbp and longer than 1.6 kbp were removed. Runs produced in our lab showed high throughput, so for each runs, only a subset of 500 k randomly picked reads was considered in this study. Raw sequencing data are available on SRA (PRJNA997989).

We selected these datasets because they were heterogeneous and allowed us to evaluate the read simulation performances under different conditions. The error rate varies from 10.59% for the Urban dataset to 17.41% for the run1 dataset. Note that the Guppy HAC model allows one to reduce the error rate from 14.45% to 11.64%. The mean read length varies from 1362 bp for the newLot dataset to 1470 bp for the Urban dataset, for which the reads were filtered out based on the read length.

The expected proportions of each taxon are known in the mock community and provided by the supplier. The taxon composition can impact the results: some taxon can be more difficult to classify than others. Several biases were observed in taxon abundances for the ZymoBIOMICS mock community. In Cusco et al. [13], a difference, more or less important depending on experiment conditions, between theoretical and actual proportions was observed. In Urban et al. [7], an over-representation of *Enterobacteriaceae* was observed across all replicates and classification methods, pointing towards a consistent *Escherichia coli* amplification bias potentially caused by skewed taxonomic specificity of the selected 16S primer pair 27F and 1492R [14]. In order to avoid abundance biases in the evaluation of read simulations, the real abundances were computed by mapping the reads with minimap2 [15] against the ZymoBIOMICS reference sequences with data of run1, run2, and run3 for the lot V1 and with the newLot, reBasecalling, and Urban datasets for the lot V2. The results are shown in Table 4.

### 4.2. Sequencing

The libraries were made with the ZymoBIOMICS Microbial Community DNA standard (Zymo Research, Irvine, CA, USA, D6305). In total, 10 ng of DNA for run1 and run2 was prepared following the Rapid 16S Rapid Amplicon sequencing kit protocol (Oxford Nanopore Technologies, Cambridge, UK, SQK-RAS201). The 16S amplicons were produced with a 25 cycles PCR program, and 64 fmol (run1) or 110 fmol (run2) of the PCR products was sequenced on a MinION flowcell R9.4.1 (Oxford Nanopore Technologies, FLO-MIN106) over 48 h. In total, 15 ng of DNA for run3 and newLot were prepared following the 16S Barcoding kit protocol (Oxford Nanopore Technologies, SQK-RAB204). The 16S amplicons were produced with a 30 cycles PCR program. In total, 20 fmol of the PCR products was sequenced on a MinION flowcell R9.4.1 (Oxford Nanopore Technologies, FLO-MIN106) over 48 h. Run3 and newLot samples were multiplexed on the same sequencing flowcell.

### 4.3. CuReSim-LoRM

#### 4.3.1. Read Simulation

**Figure 3:**
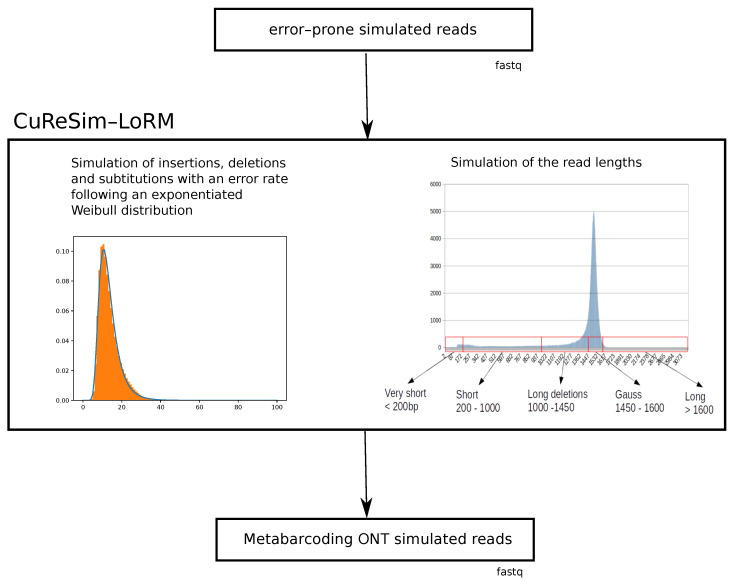
Schematic representation of the CuReSim-LoRM method.

CuReSim-LoRM is able to simulate reads with an ONT error model from reads without errors in FASTQ format obtained, for example, with Grinder [16]. Grinder allows one to simulate metabarcoding reads from reference sequences and an abundance profile to mimic a community sequencing. The schematic representation of the CuReSim-LoRM method is presented in Figure 3.

From error-prone reads, CuReSim-LoRM first introduces deletions and insertions in reads with an iterative algorithm that mostly introduces indels in the longer homopolymers. Then, substitutions are uniformly drawn. The error rate follows an exponentiated Weibull distribution with, by default, 28% of insertions, 42% of deletions, and 30% of substitutions. These values were fixed from run3 and newLot datasets as they were reproducible, and these sequencing runs were produced using the flowcell R9.4.1 and the FLO-MIN106 kit, which are the current standards for the ONT 16S metabarcoding application.

Once errors have been introduced, CuReSim-LoRM simulates the read lengths. Six categories of length have been defined in CuReSim-LoRM: (1) the Gauss category concerning read length from 1450 to 1600 bp and following a Gaussian distribution with a mean of 1500 bp and a standard deviation of 30 bp, (2) the long read category concerning reads with length greater than 1600 bp, (3) the very short reads category concerning read lengths lower than 200 bp, (4) the short read category for reads with lengths between 200 bp and 1000 bp, and (5) the long deletion category for lengths between 1000 bp and 1450 bp. Indeed, working with several ONT 16S metabarcoding datasets, we noted that a proportion of reads contained long deletions (several dozens or hundreds of bases), leading to read lengths between 1000 and 1450 bases. In CuReSim-LoRM, the size of these long deletions follows an exponential distribution. The last group, (6) the second Gauss category, was added corresponding to read length forming a second Gaussian distribution with a mean of 1100 bp and a standard deviation of 30 bp observed in some sequencing runs. CuReSim-LoRM simulates the read lengths from the percentage of these six categories, by default, 67% of the Gauss category, 2% of very short reads, 9% of short reads, 21% of long deletion category, 1% of long reads, and no secondGauss category.

At the end of the simulation, CuReSim-LoRM outputs a FASTQ file with a fixed phred score quality value, by default equal to 8, containing reads showing an ONT metabarcoding profile. The number of insertions, deletions, and substitutions introduced in each read are added at the end of the title line of the output FASTQ file.

#### 4.3.2. Training Error Models

**Figure 4:**
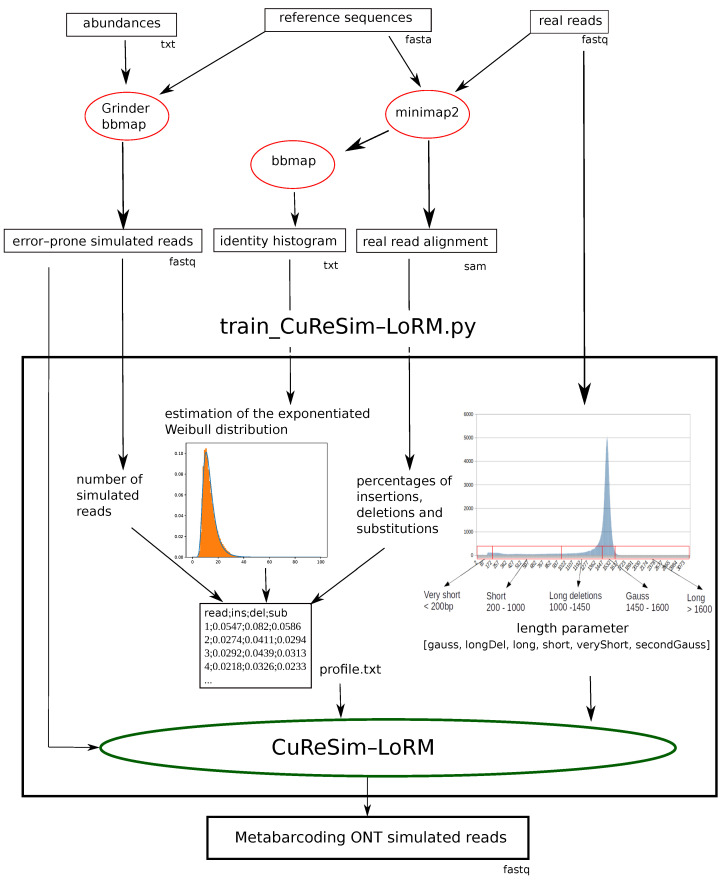
Schematic representation of the complete pipeline to train a new error model and to simulate ONT metabarcoding reads. Boxes represent input and output files; format is given under the box. Red ellipses represent external tools, and green ellipses are used for tools introduced in this study.

In order to fit different case studies and to follow future technical updates, it is possible to train a new error model from real data. A python script, train_CuReSim-LoRM.py, was developed to automate the whole process, computing the parameters and running CuReSim-LoRM. Figure 4 shows the schematic representation of the complete pipeline to train a new error model from real data and simulate ONT metabarcoding reads with CuReSim-LoRM.

The script train_CuReSim-LoRM.py requires several files in input. The FASTQ file containing the error-prone simulated reads, obtained from reference sequences and abundances, for example, with Grinder, is used to obtain *n* the number of simulated reads and as the direct input of CuReSim-LoRM. Real reads have to be mapped against the reference sequences, for example, with minimap2, to obtain a SAM alignment file. From the SAM file, the percentages of insertions, deletions, and substitutions of the real dataset are computed. bbmap [17] is used to obtain an identity percentage histogram from the alignment file. The parameters (alpha, Kappa, loc, and scale) of the exponentiated Weibull distribution of the error rate are estimated by fitting values of the identity histogram file with the fit function of the sciPy package [18]. Then, *n* values following this distribution are drawn corresponding to the error rate of the simulated reads. A profile file is generated containing *n* lines consisting of the percentages of insertions, deletions, and substitutions to be introduced in simulated reads and given in parameters to CuReSim-LoRM. The last step is to compute the parameters concerning the read length from the real dataset. The percentages of each length category are computed from the real dataset and passed to CuReSIM-LoRM as a list of six integers (gauss, longDel, longRead, short, veryShort, and secondGauss). The last category, secondGauss, is optional and provided by the user. Finally, the error-prone reads, the profile file, and the length parameter are given to CuReSim-LoRM, which outputs the simulated reads, with the ONT error profile fitting the real data. Log files are also output by resuming the estimation of parameters.

CuReSim-LoRM and train_CuReSim-LoRM are available on github https://github.com/caboche/CuReSim-LoRM/ (last accessed on 23 September 2023).

### 4.4. Simulated Datasets

#### 4.4.1. CuReSim-LoRM Datasets

Simulation of error-prone reads with Grinder and bbmap

In the first step, 88,820 reads were simulated with Grinder, with the abundances shown in “simulation” columns of Table 4 and the reference genome sequences provided by ZymoBIOMICS. The option −lb=0 was used to avoid length-bias, −cb=0 to avoid copy bias, and −rd=2000 to avoid truncating the reads with a value greater than the length of the 16S gene. The FASTA file is then converted into FASTQ with a bbmap reformat script, with qfake=8 corresponding to a phred score quality equal to 8, the quality threshold often used in ONT data analysis.

Alignment of real datasets against reference sequences

The reads of the real datasets were aligned against the reference sequences provided by the supplier, with minimap2 using default parameters. The identity histograms were obtained using bbmap with a number of bins equal to 1000.

Simulation of reads with the ONT error model

The train_CuReSim-LoRM.py script was used to obtain the error profile and length parameter for the six real datasets presented in Table 3. CuReSim-LoRM was run directly by the python script in automatic way for the run2, run3, newLot, and reBasecalling datasets. For run1, the length parameter was manually changed to [70,10,1,15,0,4] to introduce a second Gaussian distribution for 4% of the reads, and 5% of random reads were introduced with CuReSim-LoRM using the −r parameter. In the Urban run, the raw dataset was filtered to remove reads shorter than 1.4 kbp and longer reads than 1.6 kpb, so we manually modify the length parameter to [95,5,0,0,0,0] to obtain 95% of read lengths following a Gaussian distribution and 5% of reads with long deletions.

#### 4.4.2. NanoSim-H and DeepSimulator Datasets

NanoSim-H and DeepSimulator were not developed to deal with metabarcoding data and so are not able to deal directly with abundances and multi-fasta files. To counter this, each file containing a copy of the 16S gene sequence of each strain was given to Grinder to obtain a FASTA file containing the corresponding in silico amplicon. We then computed the number of reads to be generated per 16S RNA gene and per strain with the proportion of Table 4 of newLot, to obtain a total number of 88,821 reads. From each file containing the in silico amplicon and from the number of reads, the simulation was then run with both simulators. For NanoSim-H (version 1.1.0.4), three datasets were generated. Dataset1 was obtained using the −circular option, as in [10], dataset2 was generated without the −circular option, and dataset3 was obtained by training the error model with the real data of the newLot dataset and using the option −circular. We cannot test without this option because of an execution error. DeepSimulator (v1.5) was run with default settings as used previously [8].

#### 4.4.3. Evaluation Metrics

To evaluate if the simulated data mimics the real data well, several metrics were used. First, we compared the read length distribution between simulated and real data. Plots were generated using the ggplot library in R [19]. Then, simulated and real reads were first mapped against the eight reference ZymoBIOMICS sequences, and the percentage of sequencing errors and unmapped reads was obtained with a python script from the alignment file. bbmap was used to obtain the identity percentage and the standard deviation for each dataset. Reads were then mapped against the Ribosomal RNA database (rrnDB, version 5.5) containing 22,351 sequences [20] and the silva database [21] release 132 containing 95,171 sequences. A python script was used to obtain the precision and recall values for each of the three databases at the species level. Precision was computed as the number of true positives divided by the number of assigned reads and recall as the number of true positives divided by the total number of reads, with true positives corresponding to the reads assigned to one of the eight species of the zymoBIOMICS community. If simulated data mimics the real data well, the precision and recall values will be similar between real and simulated data, respectively.

## Figures and Tables

**Table 1:** Metrics obtained for simulated data compared to the real newLot dataset. The error rate value corresponds to the sequencing error rate obtained from the alignment file. The %unmapped value gives the percentage of unmapped reads against the ZymoBIOMICS reference sequences with minimap2. The %identity value gives the percentage of identity computed by bbmap from the alignment file, and the SD value is the standard deviation of the percentage of identity. The precision and recall lines give the precision and recall values from the assignment against the zymoBIOMICS reference sequences (Z) and against the rrnDB (R) and silva (S) databases. Note that the values in bold front are the best metrics obtained from simulations.

Metrics	NewLot	CuReSim-LoRM	NanoSim-H	NanoSim-H	NanoSim-H	Deep-Simulator
			Dataset1	Dataset2	Dataset3	
error rate	14.45	**14.5**	12.94	13.28	18.77	10.84
%unmapped	3.4	**5.5**	6.5	6.6	9.6	0.01
%identity	86.6	**86.6**	87.8	87.2	82.7	89.4
SD	5.2	**5**	3.9	3.2	3.4	1.5
precision_Z	1	**1**	0.99	**1**	**1**	**1**
recall_Z	0.96	**0.94**	0.92	0.93	0.9	1
precision_R	0.94	**0.91**	0.87	0.87	0.86	0.84
recall_R	0.91	**0.88**	0.82	0.83	0.8	0.84
precision_S	0.74	0.76	0.67	0.64	0.62	**0.73**
recall_S	0.72	**0.72**	0.6	0.59	0.55	0.73

**Table 2:** Metrics obtained for simulated data generated with CuReSim-LoRM compared to real datasets obtained from the alignment file. The error rate value corresponds to the sequencing error rate. The %unmapped value gives the percentage of unmapped reads against the ZymoBIOMICS reference sequences with minimap2. The %identity value gives the percentage of identity computed with bbmap from the alignment file, and the SD value is the standard deviation of the percentage of identity. The precision and recall lines give the precision and recall values from the assignment against the zymoBIOMICS reference sequences (Z) and against the rrnDB (R) and silva (S) databases. The “sim.” columns give the metrics obtained with CuReSim-LoRM simulated data, and the other columns give the metrics obtained with the respective real data.

Metrics	run1	sim.	run2	sim.	run3	sim.	reBasecalling	sim.	Urban	sim.
Error rate	17.41	17.4	16.39	16.45	14.24	14.35	11.64	11.2	10.59	10.6
%unmapped	16.8	15.76	0.7	4.9	2.6	3.9	4.5	3.7	0	1.6
%identity	84.2	84.5	84.8	85	86.8	86.8	88.8	89.3	89.8	89.9
SD	4.7	4.6	4.3	4.6	5.2	5	4.9	4.5	3.6	3.8
precision_Z	1	1	1	1	1	1	1	1	1	1
recall_Z	0.83	0.84	0.99	0.95	0.97	0.96	0.95	0.96	1	0.98
precision_R	0.94	0.92	0.96	0.93	0.95	0.94	0.93	0.91	0.94	0.92
recall_R	0.8	0.81	0.95	0.91	0.94	0.92	0.9	0.89	0.94	0.9
precision_S	0.71	0.73	0.78	0.77	0.81	0.81	0.76	0.79	0.69	0.75
recall_S	0.6	0.61	0.77	0.73	0.78	0.78	0.72	0.76	0.69	0.74

**Table 3:** Description of the sequencing datasets used in this study.

Name	#Reads	Lot	Basecaller	Error Rate (%)	Mean Length (bp)	SD (bp)	Reference
run1	2,388,682	V1	Albacore v2.1.7	17.41	1378.28	571.24	this study
run2	3,263,535	V1	Albacore v2.1.7	16.39	1464.85	144.34	this study
run3	1,108,390	V1	guppy_fast v3.4.5	14.24	1399.014	289.30	this study
newLot	1,339,249	V2	guppy_fast v3.4.5	14.45	1362.24	331.43	this study
reBasecalling	1,487,976	V2	guppy_hac v3.4.5	11.64	1364.99	381.26	this study
Urban	361,582	V2	guppy_fast v3.1.5	10.59	1470.81	25.62	[7]

**Table 4:** Proportion of the eight bacterial strains of the ZymoBIOMICS community in lot V1 and lot V2. The column «expected» gives the expected abundance as mentioned by the supplier. The following columns give the the abundances computed from real data by mapping reads against the reference sequences. The «simulation» column gives the abundances used in read simulations depending on the lot version or the dataset.

Lot V1						Expected						run1						run2						run3						Simulation					
*Lactobacillus fermentum*						0.19						0.09						0.09						0.08						0.08					
*Listeria monocytogenes*						0.16						0.16						0.16						0.14						0.14					
*Bacillus subtilis*						0.16						0.21						0.21						0.21						0.21					
*Staphylococcus aureus*						0.13						0.18						0.18						0.17						0.18					
*Salmonella enterica*						0.11						0.14						0.14						0.16						0.16					
*Enterococcus faecalis*						0.10						0.12						0.12						0.10						0.10					
*Escherichia coli*						0.10						0.10						0.09						0.11						0.12					
*Pseudomonas aeruginosa*						0.05						0.00						0.00						0.02						0.01					
**Lot V2**					**Expected**					**NewLot**					**reBasecalling**					**Simulation**					**Urban**					**Simulation**				
*Lactobacillus fermentum*					0.18					0.10					0.10					0.10					0.05					0.05				
*Listeria monocytogenes*					0.14					0.13					0.13					0.13					0.07					0.07				
*Bacillus subtilis*					0.17					0.18					0.18					0.18					0.13					0.13				
*Staphylococcus aureus*					0.16					0.18					0.18					0.18					0.09					0.09				
*Salmonella enterica*					0.10					0.15					0.15					0.15					0.30					0.30				
*Enterococcus faecalis*					0.10					0.09					0.09					0.08					0.05					0.05				
*Escherichia coli*					0.10					0.16					0.16					0.16					0.30					0.30				
*Pseudomonas aeruginosa*					0.04					0.02					0.02					0.02					0.01					0.01				

## Data Availability

Raw sequencing data are available on SRA (PRJNA997989): run1 SRR25400691; run2 SRR25400690; run3 SRR25400689; newLot SRR25400688; and reBasecalling SRR25400687. CuReSim-LoRM is developed in Java and distributed as an executable jar file not requiring any installation step. CuReSim-LoRM and train_CuReSim-LoRM.py are available on github https://github.com/caboche/CuReSim-LoRM/ (accessed on 9 September 2023) under the GNU GPL V3 licence. All the files used and generated in this study were deposited on https://doi.org/10.5281/zenodo.8199214 (accessed on 9 September 2023), including simulated reads in FASTQ format.
